# Reprogramming of pro-inflammatory human macrophages to an anti-inflammatory phenotype by bile acids

**DOI:** 10.1038/s41598-017-18305-x

**Published:** 2018-01-10

**Authors:** Marianne Wammers, Anna-Kathrin Schupp, Johannes G. Bode, Christian Ehlting, Stephanie Wolf, René Deenen, Karl Köhrer, Dieter Häussinger, Dirk Graf

**Affiliations:** 1 Department of Gastroenterology, Hepatology and Infectious Diseases, Heinrich-Heine-University Duesseldorf, Duesseldorf, Germany; 2 Biological and Medical Research Centre (BMFZ), Cluster of Excellence on Plant Sciences (CEPLAS), Heinrich-Heine-University Duesseldorf, Duesseldorf, Germany

**Keywords:** Transcriptomics, Primary biliary cirrhosis

## Abstract

Cholestasis is caused by autoimmune reactions, drug-induced hepatotoxicity, viral infections of the liver and the obstruction of bile ducts by tumours or gallstones. Cholestatic conditions are associated with impaired innate and adaptive immunity, including alterations of the cellular functions of monocytes, macrophages, NK cells and T-cells. Bile acids act as signalling molecules, affecting *lipopolysaccharide* (LPS)-induced cytokine expression in primary human macrophages. The present manuscript investigates the impact of bile acids, such as *taurolithocholic acid* (TLC), on the transcriptome of human macrophages in the presence or absence of LPS. While TLC itself has almost no effect on gene expression under control conditions, this compound modulates the expression of 202 out of 865 transcripts in the presence of LPS. Interestingly, pathway analysis revealed that TLC specifically supressed the expression of genes involved in mediating pro-inflammatory effects, phagocytosis, interactions with pathogens and autophagy as well as the recruitment of immune cells, such as NK cells, neutrophils and T cells. These data indicate a broad influence of bile acids on inflammatory responses and immune functions in macrophages. These findings may contribute to the clinical observation that patients with cholestasis present a lack of response to bacterial or viral infections.

## Introduction

Under cholestatic conditions, bile secretion and bile flow are disturbed. Healthy individuals have serum bile acid concentrations of ~3 µM, while bile acid concentrations up to ~200 µM have been found in cholestatic patients^1^. Cholestasis can be induced by the obstruction of bile ducts by tumours or gallstones, autoimmune reactions, pregnancy, drug-induced hepatotoxicity, autosomal-recessive disorders as well as liver infections. Cholestatic diseases can be forced by various causes; this condition is independent of both age and gender and results in diverse outcomes. Nevertheless, cholestatic conditions are associated with chronic inflammation of the liver. This inflammatory process is accompanied with Kupffer cell activation, accumulation of activated immune cells and results in hepatocellular apoptosis and necrosis. Other side effects of cholestasis are the proliferation of bile duct epithelial cells and activation of stellate cells, resulting in liver fibrosis^2–5^. In contrast to the cholestasis-driven immune induction, immune function in response to bacterial or viral infections is impaired in cholestatic patients. This impairment is accompanied by reduced pro-inflammatory responses of immune cells, such as monocytes, macrophages, NK cells and T cells^6–8^, associated with higher morbidity and mortality rates in cholestatic patients. Moreover, these patients exhibit higher susceptibility to bacterial and viral infections compared to healthy donors^9–12^.

Macrophages are part of the innate immune system and contribute to the activation of adaptive immunity. These cells exhibit marked functional diversity and can be separated into at least three subclasses: classically activated macrophages, wound healing macrophages and regulatory macrophages^13^. While classically activated macrophages are recruited by the first immune reaction and express high levels of pro-inflammatory mediators, regulatory macrophages induce anti-inflammatory effects. After induction, e.g., by *lipopolysaccharide* (LPS), classically activated macrophages secrete high levels of chemokines and cytokines to induce and mediate immune reactions^13,14^. Regulatory macrophages express a variety of anti-inflammatory soluble mediators, including *Interleukin-10* (IL-10), rather than pro-inflammatory chemokines and cytokines^7,15^. Bile acids reduce the LPS-induced expression of pro-inflammatory cytokines, such as IL-6, tumour necrosis factor (TNF) and IL-12 in human macrophages, while IL-10 expression remains stable. Consequently, bile acids induce a rather regulatory phenotype in macrophages^7^. Macrophages differentiate into a broad spectrum of subtypes showing characteristics of classically activated macrophages as well as regulatory macrophages, depending on cell origin, location and microenvironment^16^. Diverse subtypes of macrophages were also described for chronic diseases associated with immunity^17^. The recent results from the transcriptome and proteome profiling of macrophages showed that the division of macrophages into classically activated and regulatory macrophages cannot reflect the complex *in vivo* situation.

The composition of bile acids (BAs) in humans is dependent on diet, life-style, comorbidities and age-dependent modifications of the gut bacterial flora. The total amount of bile acids circulating in the body is called the bile acid pool and consists of the primary BAs *cholic acid* (CA) and *chenodeoxycholic acid* (CDCA) and their respective secondary BAs *deoxycholic acid* (DCA) and *lithocholic acid* (LCA)^18^. All bile acids can be conjugated with taurine or glycine. The secondary bile acid *taurolithocholic acid* (TLC) is one of the strongest agonists of *G-protein coupled bile acid receptor 1* (TGR5), which is expressed on human macrophages^19^. Nevertheless, the main bile acid transporter *Na*^+^*-taurocholate cotransporting polypeptide* (NTCP) is not detectable in human macrophages. The activation of TGR5 leads to increased intracellular cyclic AMP (cAMP) concentrations and the stimulation of two different cAMP-activated proteins: the *exchange protein directly activated by cAMP* (EPAC) and *protein kinase A* (PKA)^20,21^. The negative effects of bile acids on immune induction through TGR5 activation have been shown in macrophages and Kupffer cells^19^. With respect to blocking immune function by bile acids, PKA activation represents an essential signalling pathway^7^. Considering the current literature, it is highly likely that TGR5 plays a central role by mediating the PKA-dependent inhibitory effects of bile acids on macrophage function.

Most data concerning the modulation of immune cell activity under cholestatic conditions are derived from animal studies using *bile duct ligation* (BDL) as a model for extrahepatic cholestasis or drug-induced cholestasis. However, immune induction in humans, particularly the impact and function of cytokines and chemokines, differs from that in rodents and is not directly transferable^22^. Hence, studies with human materials are imperatively needed. Based on the fact that BAs modulate immune induction in human macrophages, we performed a transcriptome analysis to determine the impact of bile acids on the immune activation in human LPS-stimulated macrophages. The present analysis revealed an extensive influence on immune function in human macrophages by bile acids.

## Results

### Identification and mapping of differentially expressed transcripts in LPS-stimulated macrophages

**Figure 1:**
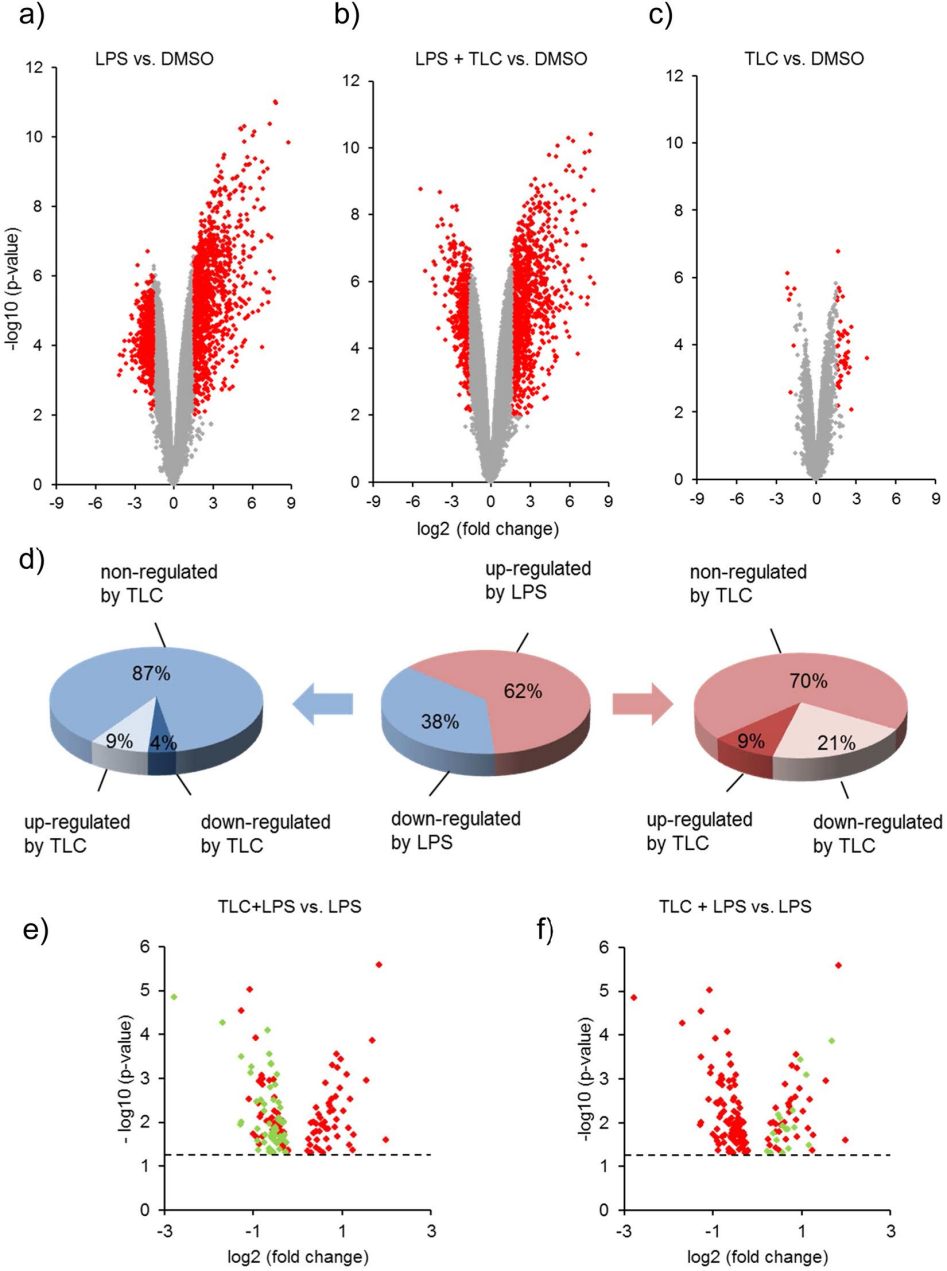
Differentially expressed transcripts identified by microarray analysis. Volcano plots indicate the differentially expressed transcripts in human macrophages after treatment with 50 µM TLC for 45 minutes and/or 10 ng/ml LPS for additional 3 h. The negative log10 transformed p-values (y-axis) were plotted against the average log2-fold change (x-axis) in gene expression. Transcripts with a p-value ≤ 0.01 and three-fold up- or down-regulated were classified as statistically significant. Significantly differentially expressed transcripts are denoted in red. (**a**) Volcano plot shows LPS-stimulated macrophages compared to control DMSO. Additionally, volcano plots of differentially expressed transcripts pre-stimulated with the conditions TLC and LPS (**b**) or TLC alone (**c**) compared to control are shown. (**d**) The central pie chart shows the LPS-affected transcripts (p-value ≤ 0.01 and a fold change ≥ 3), divided in up-regulated (red) and down-regulated (blue) transcripts. The pie charts on the right and left side show the effect of TLC on LPS-stimulated transcripts. (**e**,**f**) Volcano plots demonstrate all LPS-stimulated transcripts that were significantly modified by TLC (p-value ≤ 0.05). The dotted line indicates the -log10 of p = 0.05. (**e**) Transcripts marked in green are associated with immune regulatory functions and are down-regulated by TLC. These transcripts are also listed in Fig. 2. (**f**) Transcripts marked in green are associated with immune regulatory functions and are up- regulated by TLC. These transcripts are listed in Fig. 5.

Under cholestatic conditions, when bile acid levels are highly increased, patients suffer from an impaired immune cell function associated with increased morbidity and mortality^7,10–12^. To determine the influence of bile acids on immune induction in LPS-stimulated human macrophages, a microarray-based transcriptome analysis was performed. Human monocytes were isolated from buffy coats and differentiated to macrophages. Macrophages were incubated with 50 µM of the bile acid TLC (45 min) and 10 ng/ml LPS (3 h) prior to RNA isolation. Due to the amount of data and intrinsic variation of the data obtained in microarray experiments, statistical methods are used to systematically extract relevant information and eliminate the associated uncertainty^23^. For the identification of differentially expressed transcripts, volcano plots were used for the concurrent display of two correlated pieces of information: fold-change and t-statistic. Therefore, scatterplots of the negative log10-transformed p-values from the gene-specific t-test on the y-axis against the log2-fold change on the x-axis were prepared. Significantly modulated gene expression was defined by a p-value ≤ 0.01 and a fold-change ≥3. Characteristic gene expression profiles were depicted for LPS (Fig. 1a), LPS and TLC (Fig. 1b) as well as TLC-treated macrophages (Fig. 1c).

LPS-induced transcripts were grouped by up- and down-regulated expression. A total of 865 differentially expressed transcripts were significantly up- or down-regulated by LPS compared to control conditions (p-value ≤ 0.01 and fold-change ≥3; Supplementary Data S1). To characterize the effect of TLC on LPS-regulated transcripts, the results were monitored for significant changes in gene expression by TLC (p-value ≤ 0.05) (Fig. 1d). Among the 865 LPS-induced transcripts, 202 differentially expressed genes were significantly modified by TLC. From 540 (62%) LPS up-regulated transcripts, 50 (9%) transcripts were further up-regulated and 111 (21%) transcripts were decreased by TLC. Among the 325 (38%) transcripts down-regulated by LPS, additional TLC treatment led to an increase of 28 (9%) transcripts and further down-regulation of 13 (4%) transcripts.

For a first overview, gene products were grouped by *Gene Ontology* (GO) analysis and *Kyoto Encyclopaedia of Genes and Genomes* (KEGG) pathway mapping to sort the differentially expressed gene products according to the biological information for higher-level systemic functions. As expected, LPS affects immune-associated gene groups and pathways, e.g., cytokine and chemokine pathways, Toll-like receptor pathways as well as RIG-I-like receptor pathways (Supplementary Tables S1 and S2). Additionally, TLC modifies the transcripts belonging to several immune-associated gene groups. Nevertheless, it is not possible to differentiate between up- and down-regulated gene expression based on different systemic functions. Consequently, KEGG-pathway and GO analyses are limited in determining the impact of bile acids on LPS-regulated genes.

KEGG pathway mapping and GO-analysis showed a broad range of LPS-induced immune-associated genes additionally affected by TLC. Based on these results, functional analysis of the transcripts was obtained by using the PubMed Central or UniProtKB databases. The analysis revealed that bile acids positively or negatively regulate a broad spectrum of immune-associated genes. The gene titles and functions are described in Supplementary Table S3. From LPS-induced and TLC down-regulated transcripts, 58 genes were associated with immunity (Fig. 1e). Furthermore, from 50 transcripts up-regulated by LPS and further significantly increased by TLC, 17 genes were associated with immune function (Fig. 1f).

In conclusion, 23.3% of the 865 differentially expressed transcripts by LPS were identified as significantly modified by TLC, and overall, 75 transcripts were associated with immunity.

### Down-regulation of LPS-stimulated genes by TLC

**Figure 2:**
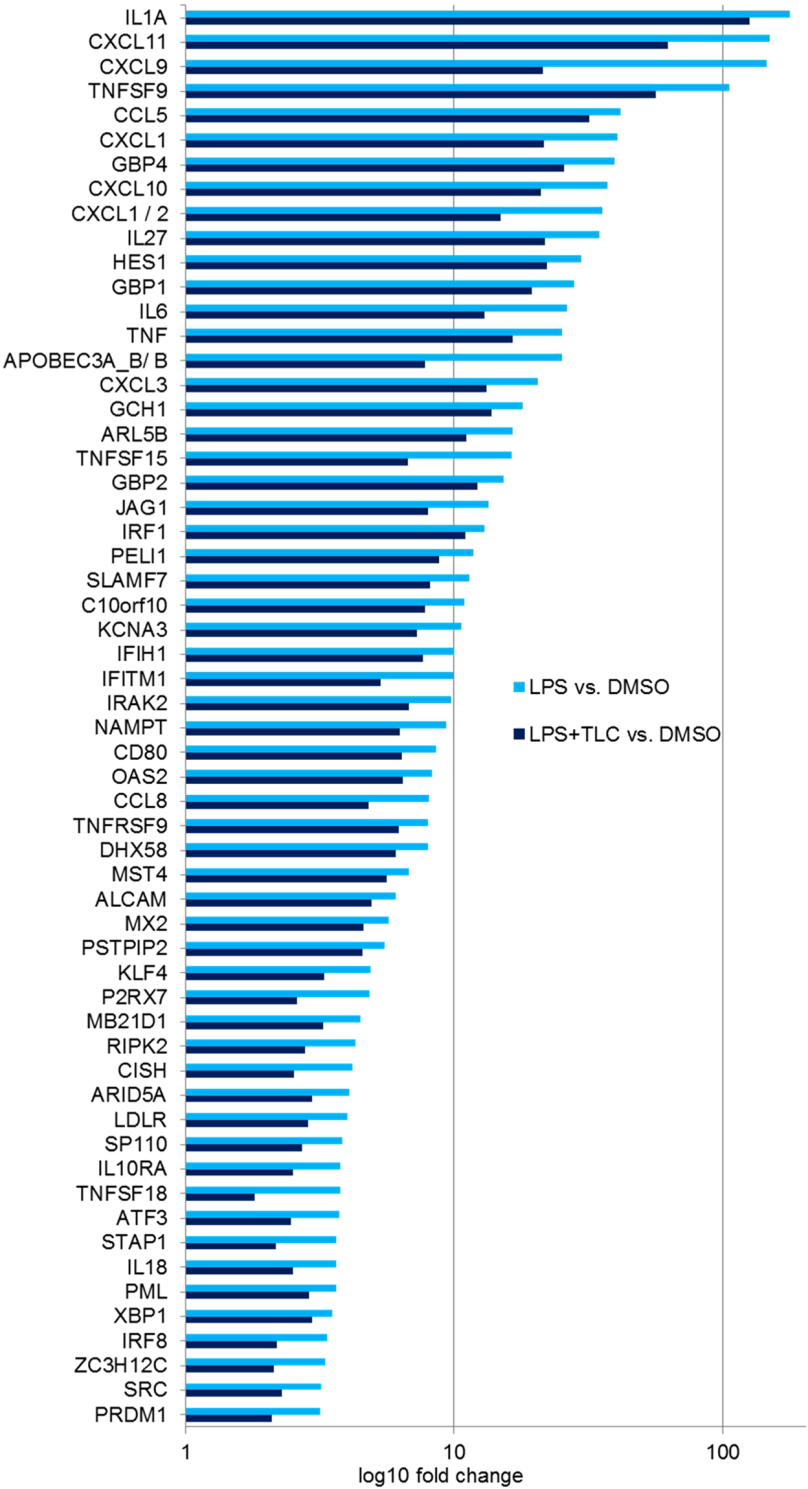
LPS-induced immune regulatory genes down-regulated by TLC. Selected immune associated genes significantly increased by LPS (fold ≥ 3 and p-value ≤ 0.01) and decreased by TLC (p-value ≤ 0.05). The vertical axis shows the name of expressed genes and the horizontal axis displays the –Log10 (fold change). Transcripts are classified as having immune regulatory properties by considering information about gene and protein function (http://www.ncbi.nlm.nih.gov or http://www.uniprot.org/).

A total of 58 genes, significantly up-regulated by LPS (fold ≥ 3 and p-value ≤ 0.01) and down-regulated by TLC (p-value ≤ 0.05), could be associated with immune function as early immune induction, chemotaxis and the activation of other immune cells (Fig. 2). Several genes directly act against pathogens or modulate intracellular signalling pathways associated with immunity in macrophages.

**Figure 3:**
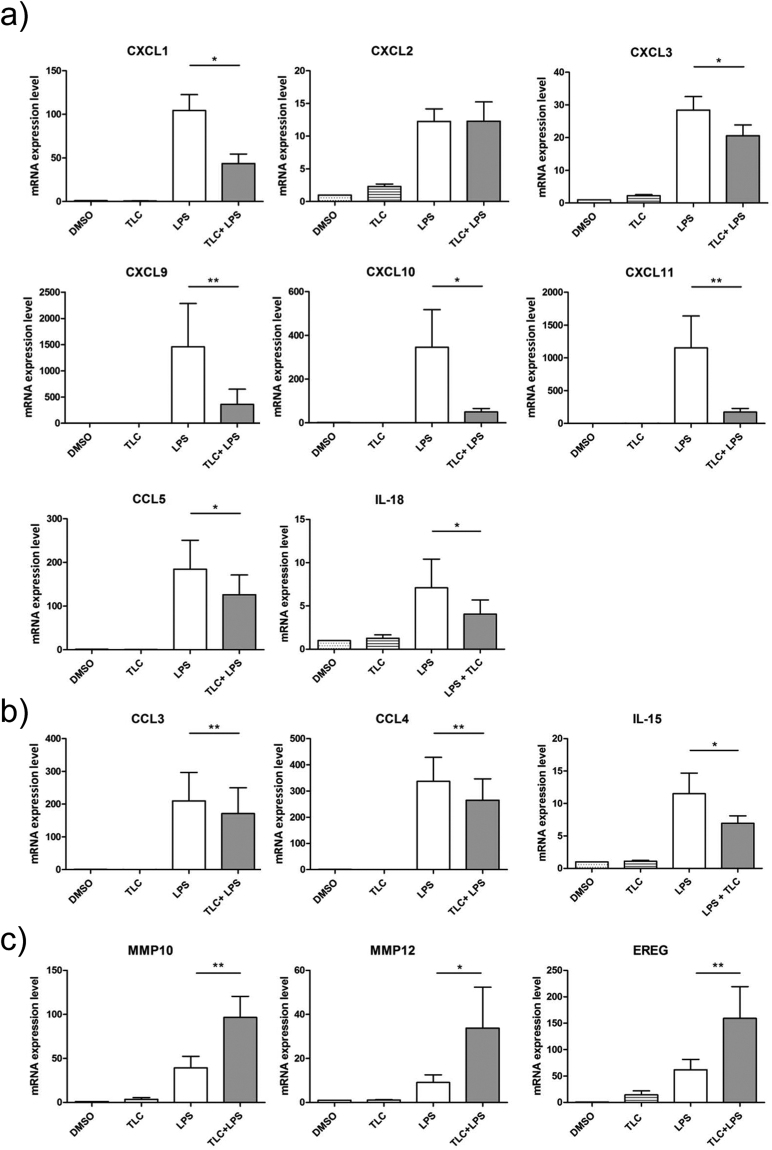
Verification of transcriptome analysis by qPCR. Primary human macrophages are pre-incubated with 50 µM TLC for 45 min, and where indicated, LPS (10 ng/ml) was added for another 3 h before the cells were lysed for RNA preparation. Levels of gene expression are determined by qPCR using specific human primers and HPRT1 as a housekeeping gene. Relative mRNA levels are acquired and compared with the control (DMSO), which is set to 1. Data are presented as the means ± sem (n = 8). *P-value ≤ 0.05; **p-value ≤ 0.005. (**a**) Expression levels of LPS-stimulated CXCL1, CXCL2, CXCL3, CXCL9, CXCL10, CXCL11, CCL5 and IL-18 are significantly reduced by TLC. (**b**) TLC blocks expression of LPS-stimulated CCL3, CCL4 and IL-15. (**c**) LPS-induced MMP10, MMP12 and EREG expression levels are further increased by TLC.

**Figure 4:**
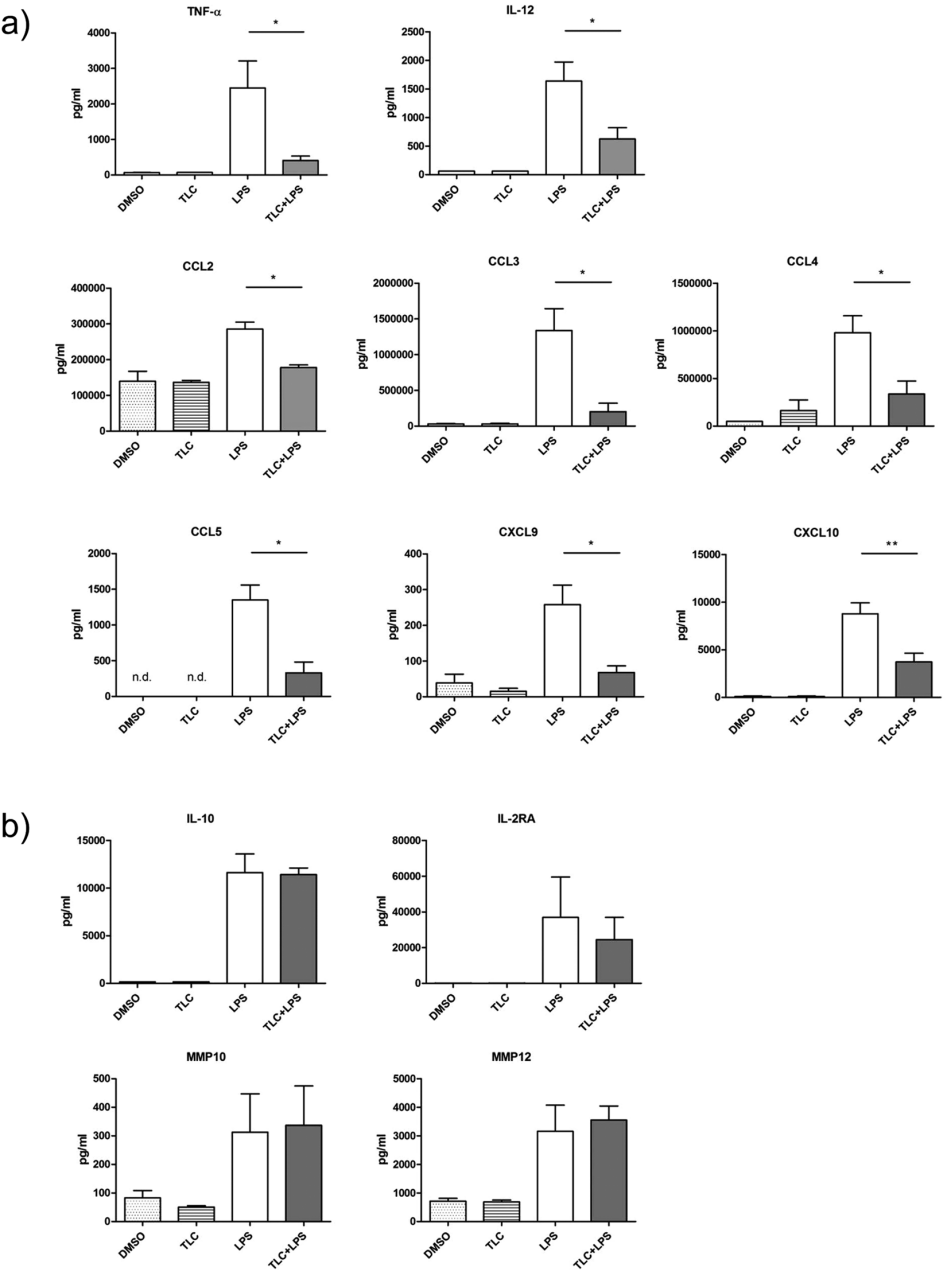
LPS-induced protein expression of different chemokines in presence or absence of TLC. Primary human macrophages are pre-incubated with 50 µM TLC for 45 min or were left untreated, and when indicated, LPS (10 ng/ml) was added for another 24 h before the supernatant was collected. (**a**) Supernatants from macrophages were analysed via Luminex Technology. Secreted LPS-induced chemokines are significantly down-regulated by TLC. (**b**) Supernatants from macrophages were analysed via Luminex Technology and ELISA and expression of IL-10, IL-2RA, MMP10 and MMP12 were detected. Data are presented as the means ± SEM (n = 4). *P-value ≤ 0.05.

A first group of significantly LPS-induced genes, which are down-regulated by TLC, include IL-1A, IL-6 and TNF. These cytokines are associated with early immune induction, as they show pyrogenic effects and induce acute phase responses. A broad spectrum of LPS-induced genes, reduced by TLC (KCNA3, TNFSF9, TNFSF18, CD80, TNFRSF9 and ALCAM), is involved in the activation, differentiation and migration of monocytes, macrophages, NK cells, neutrophils and T-cells. TLC regulates a variety of chemokines and cytokines, such as CXCL1, CXCL2, CXCL3, CXCL9, CXCL10, CXCL11, CCL5, CCL8, IL-6, IL-18 and IL-27, which are of particular importance for immune cell migration and activation. To verify the results of the transcriptome analysis, quantitative real-time PCR (qPCR) was performed. Differentiated macrophages were incubated with 50 µM TLC (45 min) and 10 ng/ml LPS (3 h). Interestingly, in the microarray analysis, the chemokines CXCL1 and CXCL2 are both significantly highly expressed. The regulation of CXCL2 by TLC in LPS-stimulated macrophages could not be determined by qPCR. Therefore, TLC most likely affects the expression of CXCL1 but not of CXCL2 since qPCR revealed a ten-fold stronger induction of CXCL1 expression by LPS compared to CXCL2 (Fig. 3a). The LPS-induced expression of CXCL1, CXCL3, CXCL9, CXCL10, CXCL11, CCL5 and IL-18 demonstrated a bile acid-dependent reduction (Fig. 3a). At the protein level, we also demonstrated the down-regulation of LPS-induced chemokines in the presence of TLC (Fig. 4a).

The functions of chemokines are multifarious and several chemokines promote the same or a similar effect on immune cell activation. LPS-induced CCL3, CCL4 and IL-15 expression promote similar effects on immune cell activation and migration, such as CCL5 and IL-18^24^. When considering the microarray data, CCL3, CCL4 and IL-15 were not significantly changed. Nevertheless, qPCR demonstrated that LPS-dependent expression of these genes is significantly reduced by bile acids (Fig. 3b) as well as CCL3 and CCL4 at the protein level (Fig. 4a). Interestingly, TLC modulates LPS-induced genes that interact with viral or bacterial pathogens and are grouped together. IFITM1, APOBEC-3A/B, GBP1, GBP2, MX2, OAS2, MB21D1, IFIH1/MDA5, DHX58 and ARL5B cause an anti-viral effect and are down-regulated by TLC. Genes with key roles in phagocytosis and autophagy, such as IRF-8, C10 or f10, LDLR and NAMPT are decreased by TLC. Several bile acid-regulated genes are involved in the signalling pathways of pattern recognition factors or other receptors for pathogens (STAP1, XBP1, KLF4, PML, PELI1, MST4, RIPK2, JAG1 and HES1). In addition, transcripts correlated with chemokine and cytokine signalling pathways, such as IRAK2, IRF-1, SRC and IL10RA. are down-regulated by TLC.

TLC-down-regulated transcripts, such as P2RX7, TNFSF15, GCH1, ARID5A, ATF-3, GBP4, SP110, CISH, PRDM1, SLAMF7, PSTPIP2 and ZC3H12C, affect nitric oxide (NO) production, cytokine stability as well as IFN production.

Various LPS-stimulated genes involved in immune induction are abolished by TLC. These genes could be clustered in different relevant immune induction groups and showed an enormous impact of bile acids on the functions of human macrophages.

### Up-regulation of LPS-stimulated genes by TLC

**Figure 5:**
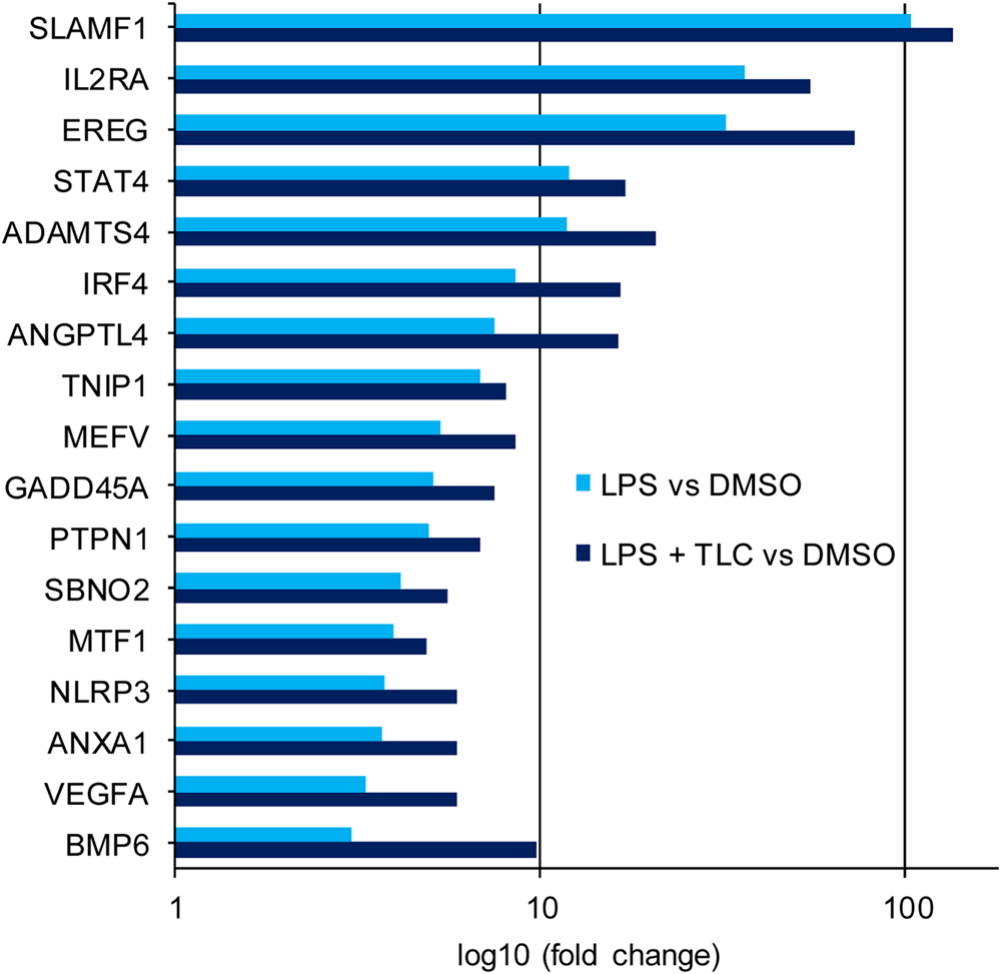
LPS-induced immune regulatory genes up-regulated by TLC. Selected immune associated genes significantly increased by LPS (fold ≥ 3 and p-value ≤ 0.01) and further triggered by TLC (p-value ≤ 0.05). The vertical axis shows the name of expressed genes and the horizontal axis displays the –Log10 (fold change). Transcripts are classified as having immune regulatory properties by considering information on gene and protein function (http://www.ncbi.nlm.nih.gov or http://www.uniprot.org/).

A small portion of the expressed genes (9%) was up-regulated by the inflammatory mediator LPS and showed an additional strong increase in gene expression after stimulation with TLC (Fig. 1d). Immunologically relevant transcripts were further estimated (Fig. 1f). Interestingly, the differentially expressed genes are involved in immunological processes, such as wound healing, cell differentiation and particularly anti-inflammatory cell signalling. LPS-induced EREG expression is significantly higher in response to additional treatment with TLC (Figs 3c and 5). EREG is a member of the *epidermal growth factor* (EGF) family and regulates the proliferation, differentiation and function of numerous tissues in humans^25^. Another growth factor activated by LPS, which is even more induced by TLC, is VEGFA, a mediator of angiogenesis and endothelial proliferation^26^. Since liver tissue is highly damaged under cholestatic conditions, the induction of endothelial growth factors and cell differentiation factors could protect the liver from additional inflammation-mediated damage. Depending on their subtypes, macrophages have been associated with wound healing, fibrosis, insulin sensitivity and immunoregulatory functions. These subtypes express ligands, such as TGF-β1, PDGF, VEGF and various *matrix metalloproteinases* (MMPs) that regulate myofibroblast activation and the deposition of extracellular matrix components^16^. Several MMPs participate in the differentiation of monocytes to macrophages. LPS-induced macrophage-specific MMP10 and MMP12 mRNA expression is significantly up-regulated in response to TLC (Fig. 3c), while the expression at the protein level is constantly compared to LPS stimulation (Fig. 4b). Thus, metalloproteinases control the balance between the beneficial effects of acute inflammation and the detrimental effects of chronic inflammation through the control of macrophage differentiation, migration and function. Furthermore, ADAMTS-4, which is up-regulated by TLC (Fig. 5), has been described to promote the differentiation of monocytes into macrophages and induce the invasion of macrophages *in vitro*^27^.

Additionally, bile acids boost the expression of a number of anti-inflammatory genes, such as ANXA1, ANGPTL4, IRF4, MEFV, PTPN1, SBNO2, SLAMF1 and TNIP1^28–31^. Additionally, BMP-6, a member of the transforming growth factor (TGF)-β superfamily, which is a negative regulator of the immune system, is significantly up-regulated at the mRNA level in the presence of TLC (Fig. 5) ^32,33^.

While GADD45A is involved in chemotactic responses, some expressed genes, such as IL-2RA, STAT4, MTP1 and NLRP3, lead to pro-inflammatory gene expression^34–37^. However, at the protein level, IL-2RA is not significantly differently regulated under the co-incubation of LPS and TLC compared to incubation with LPS alone (Fig. 4b). The induction of IL-2RA at the protein level is detectable but stable under co-incubation of LPS and TLC compared to incubation with LPS alone (Fig. 4b).

### Bile acid-dependent signalling pathways

**Figure 6:**
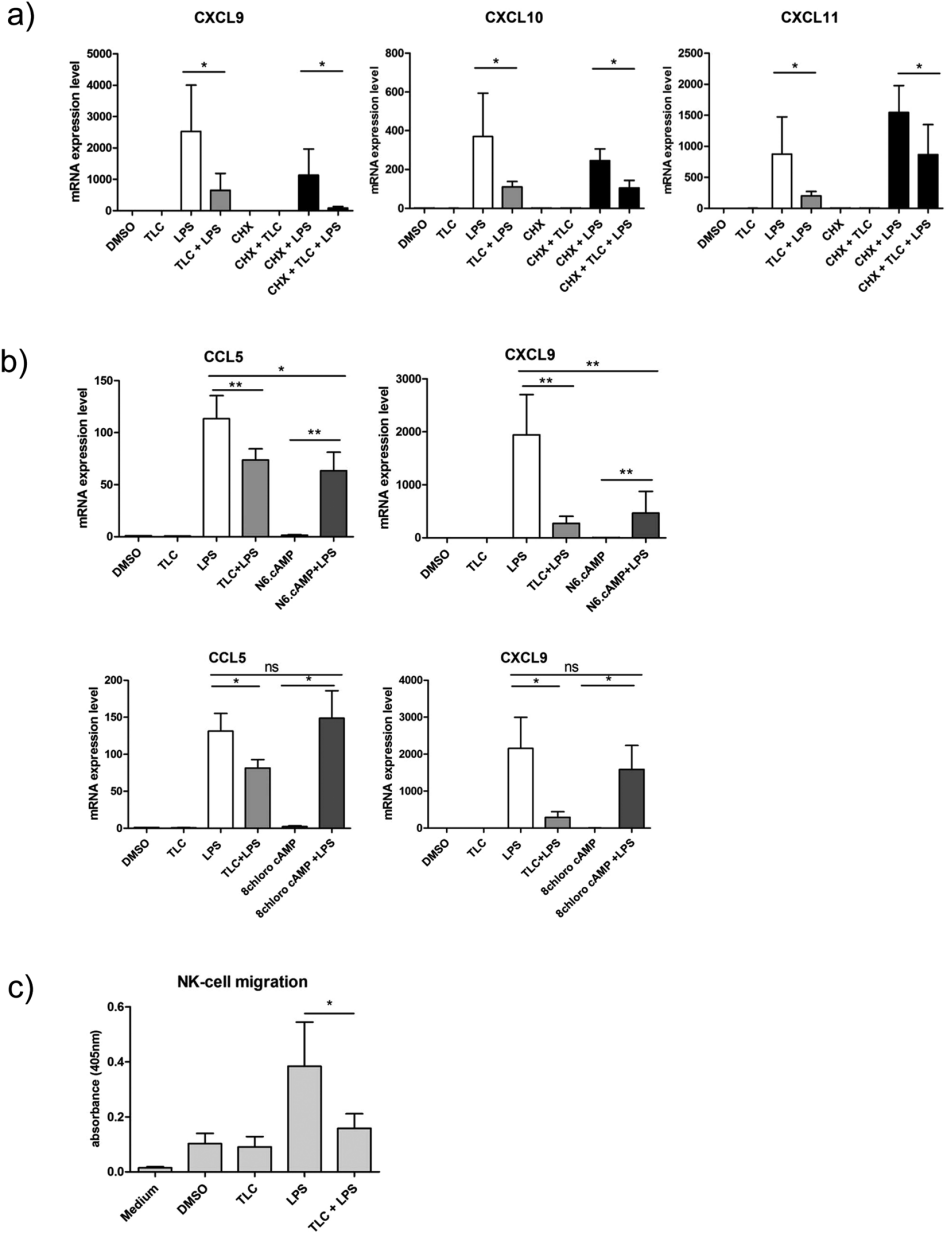
TLC influences gene expression by activation of TGR5 and PKA and NK cell migration. (**a**) TLC-regulated mRNA expression is independent of *de novo* protein synthesis. Macrophages were pre-incubated with cycloheximide (1 h; 3.55 µM) prior to stimulation with TLC and/or LPS. RNA was isolated and expression levels of CXCL9, CXCL10 and CXCL11 were determined by qPCR. (**b**) Determination of bile acids signalling through PKA- or EPAC-signalling pathways with PKA- and EPAC-specific cAMP analogues. Macrophages were pre-incubated with N6.cAMP or with 8chloro cAMP (45 min; 100 µM) and stimulated with LPS thereafter. The mRNA was isolated and qPCR for CCL5 and CXCL9 was performed. (**c**) Blockade of macrophage-dependent NK cell migration by TLC. Isolated NK cells were stimulated with supernatants of macrophages after stimulation with or without TLC and LPS. Migrated human NK cells were collected, and the cell viability was analysed by MTT. The measured absorbance is 450 nm. Data are presented as the means ± sem (n = 7). *P-value ≤ 0.05.

Several published studies suggest that bile acids can activate TGR5 in the liver, and this G-protein coupled receptor is also expressed on macrophages^7^. To identify the impact of TGR5 on the modulatory effects of bile acids, we first determined if the regulatory effect of bile acids to LPS-induced transcription depends on the *de novo* translation of a specific protein. To this end, macrophages were treated with the translation inhibitor cycloheximide (CHX; 3.55 µM; 1 h) prior to incubation with TLC and LPS. In presence of cycloheximide, TLC down-regulates the LPS-dependent mRNA expression of CXCL9, CXCL10 and CXCL11 (Fig. 6a). Consequently, the anti-inflammatory effect of bile acids is independent on the *de novo* translation of an effector protein.

TGR5 activation by bile acids leads to increased intracellular cAMP levels and the activation of PKA and EPAC. To determine if the effect of bile acids on chemokine expression depends on the PKA or EPAC signalling pathway, primary human macrophages were incubated with specific cAMP analogues. Macrophages were stimulated with either N6-cAMP (100 µM), which selectively activates PKA, or the EPAC-specific activator 8-chloro cAMP (100 µM) for 45 min prior to treatment with LPS. No significant changes in LPS-induced CCL5 and CXCL9 expression in the presence of the EPAC activating cAMP analogue 8-chloro cAMP were detectable, whereas the N6-cAMP analogue clearly inhibited LPS-induced CCL5 and CXCL9 expression (Fig. 6b). These results suggest that the impairment of LPS-induced chemokine expression by TLC is dependent on PKA activation. The receptor TGR5 might be the mediator of anti-inflammatory TLC effects on human macrophages^19^.

### Bile acids inhibit macrophage dependent NK-cell migration

We demonstrated that TLC blocks the expression of LPS-induced chemokines (Figs 3 and 4), which are involved in the migration of immune cells. To test the hypothesis that TLC abolishes LPS-induced, macrophage-dependent NK-cell chemotaxis, a migration assay was performed. Isolated human NK cells were incubated (18 h) with the supernatants of human macrophages, pre-treated with or without TLC, and further stimulated with LPS (24 h). Cell viability after bile acid stimulation was determined using the MTT assay. LPS-induced, macrophage-dependent NK cell migration was blocked to the control levels when macrophages were pre-incubated with TLC (Fig. 6c).

## Discussion

Under chronic cholestatic conditions, the normal liver architecture is destroyed, and fibrosis is induced, leading to hepatic dysfunction and portal hypertension. In addition to the direct damage of hepatocytes by high bile acid concentrations, the tissue is further damaged by dysfunctional immune cells, pro-inflammatory cytokines and chemokines^38,39^. The current study substantiates the previous data that bile acids impaired the early immune response by down-regulation of IL-6, TNF, IL-12 and IL-1α in blood-derived macrophages and Kupffer cells^7,19^. Moreover, various pathways are modulated by bile acids and mediate the interactions of several immune cells with pathogens, autophagy, phagocytosis and intracellular pro-inflammatory signalling in macrophages.

The impact of macrophages on the activation of numerous immune cells, such as NK cells, is dependent on cellular crosstalk and the cytokine microenvironment^40^. Activated NK cells in turn boost macrophage function, thereby inducing phagocytosis^3^. Under cholestatic conditions, the function and role of NK cells remains unclear and controversial. Experiments with BDL animals demonstrated the activation of a specific number of NK cells, leading to Kupffer cell stimulation and cytokine expression^3^. While the present study correlated NK cell activation with the suppression of cholestatic liver injury, other groups associated NK cell activation with liver damage^5^. However, LPS-induced cytokines, such as IL-12, IL-18 and IL-15, are associated with NK cell activation and significantly diminished by TLC. On the one hand, TLC abolished CCL3, CCL4 and CCL5 expression, which is correlated with the cytolytic activity of NK cells^24^, while on the other hand, TLC blocked expression of ligands for CXCR3, such as CXCL9 - CXCL11, which are mediators of NK-cell chemotaxis^22,41^. These findings are consistent with the observation that macrophage-dependent NK cell migration is inhibited in response to TLC (Fig. 6c). We hypothesized that macrophage-dependent NK cell activation and that migration is impaired by bile acids under acute infection conditions.

In contrast to NK cell function in cholestasis, neutrophils play a prominent role in cholestatic liver injury. After bile duct ligation in animal models, neutrophils are recruited to the liver and induce reactive oxygen species and protease release, resulting in liver parenchyma damage^2^. Neutrophils migrate in response to a broad spectrum of chemokines, including CXCL1 and CXCL3^22^. The down-regulation of neutrophil-attracting factors by bile acids, as demonstrated by transcriptome analysis, could result in diminished neutrophil function under acute infection (Figs 2 and 3a). In addition to neutrophils, CXCL1 and CXCL3 affect the migration of macrophages. A reduction of these gene products by bile acids in LPS-stimulated macrophages could result in the abolishment of macrophage migration triggered by, e.g. Kupffer cells, at the source of infection.

The early innate immune induction is followed by adaptive responses, such as T cells activation. Naïve T cells are found in secondary lymphatic organs or in peripheral circulation. Under infectious conditions, naïve T cells are activated by binding antigen-presenting cells, such as macrophages. T cell-activating factors on the surface of macrophages (CD80, TNFSFs and ALCAM) are significantly reduced by bile acids^42,43^. Activated T cells migrate and differentiate in response to chemokines and cytokines, which are expressed by human macrophages^42,44^. LPS-induced expression of CXCL9, CXCL10, CXCL11, IL-18, IL-6 and IL-27 by macrophages is normally involved in T cell differentiation, activation and migration. Down-regulation by TLC could result in suppressed T cell function and impaired adaptive immunity under cholestatic conditions. The ability of macrophages to react to different stimuli with a wide range of diverse responses makes these cells important regulators of immune responses. Moreover, an imbalance in macrophage differentiation has a major impact on the progression and resolution of many chronic diseases^16^.

The impact of TLC could be demonstrated by enhancing a number of expressed genes, such as ADAMTS-4, BMP-6, VEGFA or EREG, at the mRNA level. These proteins are involved in immunological processes responsible for wound healing and anti-inflammatory cell signalling (Fig. 5) and reflect a characteristic feature of regulatory macrophages. EREG plays a role in skin inflammation and cutaneous wound healing^45,46^. BMP-6 belongs to the TGF-β superfamily and is a potent immune regulator reported to have an anti-proliferative effect on B and T cells^32,33^. Additionally, LPS-induced expression of macrophage-specific MMP10 and MMP12 is triggered in the presence of TLC on mRNA level. However, at the protein level, both MMPs are not induced by TLC compared to LPS alone, but protein expression is at least not blocked by TLC, resulting in an imbalance between high levels of MMPs on the one hand and low protein levels of diverse chemokines on the other hand in the presence of TLC.

Macrophage-specific MMP10 plays an important role in mediating migration and invasion of macrophages^47^ and MMP12 assists the regulation of acute inflammatory responses^48^. Under these conditions, macrophages not only fail to produce inflammatory mediators, but also modulate immune responses through the secretion of immunosuppressive/anti-inflammatory cytokines (Supplementary S3) ^49^. In contrast, some pro-inflammatory LPS-induced genes increased in response to TLC. The up-regulated expression of IL-2RA was not comprehensible at the protein level induction (Fig. 4b) but was at least stable under co-incubation from TLC and LPS compared to LPS alone. A reason for the elevated expression of IL-2RA and STAT4 by TLC might be autocrine signalling as a form of cell signalling in which a cell secretes chemical messenger that bind to autocrine receptors on the same cell, leading to changes in the cell^34,50^. Pro-inflammatory genes, such as MTF1 and NLRP3 are also up-regulated in the presence of TLC. The enhanced expression could be caused by a dysregulation of negative feedback loops, which are normally activated by LPS and potentially diminished by bile acids. Interestingly, a study demonstrated that the LPS-induced effect on NLRP3 was stronger in classic activated macrophages (M1), whereas there was no effect in alternatively activated macrophages (M2)^36^. The increased expression of the aforementioned LPS-induced genes could lead to a further manifestation of the anti-inflammatory effects of bile acids in macrophages.

This result confirms our suggestion that there is not a rigid differentiation from the M1 to M2 macrophage phenotype but rather smooth transitions between specific subtypes exist with changes in cytokine patterns depending on the cellular microenvironment under cholestatic conditions.

TLC down-regulates the expression of signal elements, such as STAT1, JAG1 and XBP1, in macrophages, associated with pro-inflammatory cellular functions and connected with Toll-like receptor pathways or other pathogen-activated signalling pathways (NOTCH and NOD)^51,52^. Moreover, cytokine/chemokine receptor pathways, cytokine stability and cytokine release are impaired by bile acids in LPS-stimulated macrophages. Additionally, TLC interferes with signal elements, which are part of the IL-1 and IL-10 signalling pathways. Bile acids affect the response of macrophages to pathogen recognition and inflammatory extracellular signals, leading to a misbalance of immune induction in macrophages.

TLC also modifies TLR signalling pathways in human macrophages. The TLR-4-mediated activation of genes, which are directly associated with pathogen elimination, are down-regulated by TLC in LPS-stimulated macrophages. Transcripts, such as APOBEC-3A, MX2 and IFIH1/MDA5 (Fig. 2), are negative regulators of the viral replication cycle and bacterial growth. MX2 constrains viral replication and genome integration to the host genome, and APOBEC-3A causes the hypermutation of viral nucleic acids^53^. Additionally, cytoplasmic sensors of viral RNA and DNA, such as MDA5, are decreased by bile acids^54^. Moreover, TLC reduces the expression of LPS-induced factors associated with autophagy and phagocytosis, such as LDLR and IRF8 (Fig. 2) ^55,56^. The effects of bile acids could be associated with the described reduced pathogen clearance in BDL mice and cholestatic patients^9–12^.

Furthermore, the modulatory effect of TLC on LPS-induced macrophages not only converts classically activated macrophages to regulatory macrophages but rather stimulates differentiation to a macrophage subtype showing properties of both macrophage types. Current studies demonstrated that macrophages not only differentiate to classically activated or alternatively activated macrophages but to diverse subtypes of macrophages. This diversity is independent of cellular origin, location and microenvironment, and the macrophage changes have an enormous impact on chronic diseases^16,17^. Therefore, the results of the transcriptome analysis can support a cholestasis specific macrophage subtype, associated with reduced pro-inflammatory response, enhanced or at least stable expression of anti-inflammatory cytokines and increased regulatory and wound healing properties.

It is most likely that these results are also transferable to Kupffer cells. These specialized macrophages, located in the liver, express the same setting of receptors for LPS (CD14, TLR4) and bile acids (TGR5, PKA)^19,57^. Additionally, bile acids also decrease early immune induction, measured by LPS-induced TNF, IL-6, IL-1α and IL-1β expression in Kupffer cells. This regulation depends on the TGR5-PKA signal pathway^19^, which is also correlated with the immune regulatory properties of bile acids in human macrophages in the present study (Fig. 6b).

**Figure 7:**
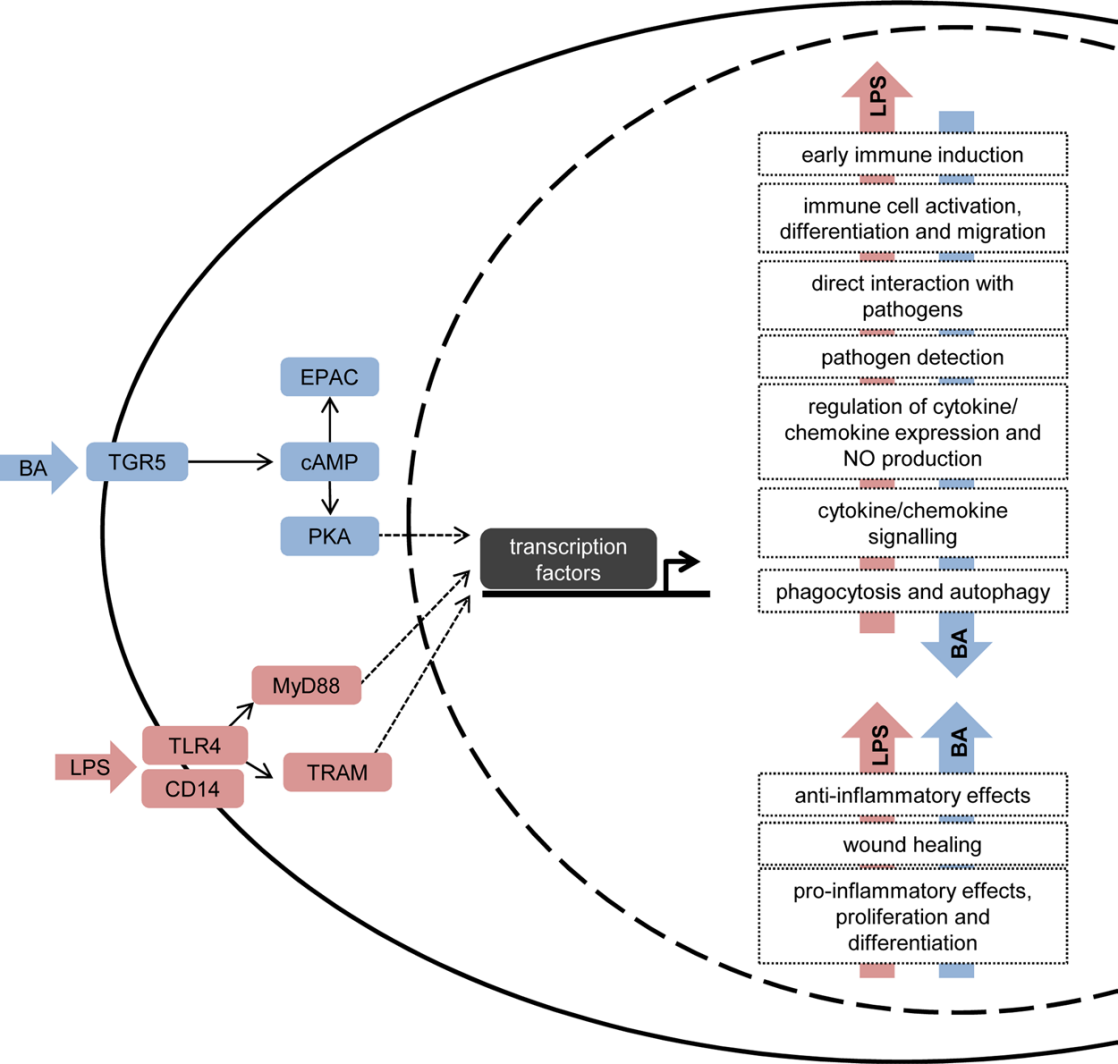
Bile acids modulate a broad spectrum of LPS-induced pro-inflammatory and anti-inflammatory genes. LPS activates the TLR4 receptor, which signals through MyD88 and TRAM, followed by the activation of different transcription factors, resulting in expression of pro-inflammatory and anti-inflammatory genes. Bile acids affect the expression of these pro-inflammatory (down-regulation shown with a red arrow), anti-inflammatory and regulatory (up-regulated shown with a blue arrow) genes by activating the TGR5 bile acid receptor and cAMP-dependent PKA activation on human macrophages.

Under cholestatic conditions, patients exhibit higher morbidity and mortality rates as a consequence of bacterial and viral infection^9–12^. Previous studies have shown that bile acids affect the pro-inflammatory cytokine expression in human blood derived macrophages and Kupffer cells, while the expression of the anti-inflammatory mediator IL-10 is constant^7,19^. The present results of the transcriptome analysis revealed strong inhibitory properties of the bile acid TLC on LPS-induced immune induction in human macrophages. The modulatory effect of bile acids on LPS-stimulated macrophages impairs the expression of gene products associated with early immune induction, phagocytosis, immune cell activation and chemotaxis (Fig. 7). These results suggest that the development of a specific macrophage subtype is based on the patient’s cholestatic phenotype.

In summary, TLC stimulation resulted in an imbalance between abolished pro-inflammatory genes on the one hand and at least stably expressed anti-inflammatory and regulatory genes or genes involved in wound healing. This modified gene profile resulted in a phenotype of regulative acting macrophages, likely representing a cholestasis-specific macrophage subtype. The modulatory effect of TLC on LPS-induced macrophages not only converts classically activated macrophages (M1) to regulatory macrophages (M2b) but also stimulates the differentiation to a macrophage subtype showing properties of both macrophage types with changes in cytokine patterns. These effects may result in a reduced clearance of infected cells and pathogens, as observed in cholestatic patients. However, if activated immune cells contribute to liver damage, the reduced immune induction after acute infection under cholestatic conditions could also represent a partially protective mechanism of bile acids against fulminant liver damage^2,5^.

## Material and Methods

The present study was performed according to the guidelines of the Declaration of Helsinki, and written informed consent was obtained from each blood donor. The donors consented to the use of their blood samples for research purposes as part of the blood donation at the Heinrich Heine University of Düsseldorf, Blood Transfusion Service. Research on human blood samples as presented in the present study was approved through the ethical review committee of the Medical Faculty of the Heinrich Heine University Düsseldorf (approval number 3891).

### Antibodies and chemicals

Bile acids (TLC), cycloheximide, 8(4-Chlorophenylthio)-2′-O-methyladenosine 3′,5′-cyclic monophosphate monosodium hydrate (8chloro cAMP) and N6-Benzoyladenosine-3′,5′-cyclic monophosphate sodium salt (N6 cAMP) were obtained from Sigma-Aldrich (St. Louis, USA).

### Separation and differentiation of primary human macrophages

Peripheral blood mononuclear cells (PBMCs) were isolated from human blood (healthy volunteers, male) using buffy coats obtained from the Heinrich-Heine-University of Düsseldorf, Blood Transfusion Service, by Ficoll Plaque gradient centrifugation. CD14-positive cells were separated by antibodies labelled with magnetic beads, according to the manufacturer’s instructions, using human CD14 MicroBeads, LS columns, for QuadroMACS (Miltenyi Biotec, Bergisch Gladbach, Germany) and 3 × 10^6^ cells/well were cultured for 9 days. RPMI medium supplemented with 10% FCS, 100 U/ml penicillin, 100 mg/ml streptomycin and 25 ng/ml human M-CSF was used to differentiate these cells into macrophages. Medium volume was increased every 3rd day by 30% inclusive of freshly added M-CSF. Cells were grown at 37 °C in 5% CO_2_. Primary human macrophages were pre-incubated for 45 min with 50 µM TLC, and where indicated, LPS (10 ng/ml) was added for another 3 h. For controls, macrophages were incubated with DMSO, TLC and LPS for the same duration.

### NK cell separation and cell-migration assay

Blood samples were taken from healthy donors and loaded on Ficoll density gradient to purify the lymphocyte population. NK cells were prepared by magnetically labelled beads, according to manufacturer’s instructions, using human NK cell selection MicroBeads, LS columns, for QuadroMACS (Miltenyi Biotec, Bergisch Gladbach, Germany). RPMI medium supplemented with 5% FCS, 100 U/ml penicillin, 100 mg/ml streptomycin was used to culture NK cells. NK cells (3 × 10^6^) in 100 μL were loaded into each Transwell filter (5-μm pore filter Transwell, 24-well cells clusters; Corning, Corning, Kaiserslautern, Germany). The filters were then plated in each well containing 600 μL supernatants from TLC-pre-treated and LPS-stimulated macrophages (24 h). After 18 h of incubation at 37 °C and 5% CO_2_, the upper chambers were removed, and cells in the bottom chamber were collected and analysed by MTT (3-(4,5-dimethylthiazol-2-yl)-2,5-diphenyltetrazolium bromide). The detected tetrazolium reduction is proportional to the number of presented viable cells.

### MTT Assay

Migrated cells and a medium control without cells were transferred at a volume of 100 μl per well in a 96-well plate, and 20 µL of the 10 mM MTT stock solution was added to each well and subsequently incubated at 37 °C for 4 hours or overnight. The supernatants were carefully removed after centrifugation at 300 rcf and 4 °C for 5 min. DMSO (50 μl) was added to the wells before incubation on a shaker in the dark for approximately 20 min. The absorbance was quantified at 570 and 650 nm as a reference wavelength.

### ELISA Assay

The cells were pre-incubated with TLC followed by stimulation with LPS. After 24 h, the supernatants were collected and MMP10 and MMP12 concentration was measured by ELISA (RayBiotech; Cohersion Biosciences, London, UK) according to the manufacturer’s instructions. Protein level was determined by using a standard curve with MMP10 and MMP12. The absorbance was measured at 450 nm by using the Thermo Scientific Multiskan spectrum.

### Multiplex Immunoassay

Cells were pre-incubated with TLC, followed by stimulation with LPS (24 h). Cell culture supernatant was collected under sterile conditions, followed by centrifugation (5 min, 4 °C, 7.500 rpm). Aliquots were pre-cooled at −20 °C and stored in −80 °C until quantitative mediator measurement. Analysis of cytokine concentration in supernatants was performed by using Luminex Technology (Austin, Texas, USA) and the human cytokine magnetic 25-Plex Panel (Novex Live Technologies, Karlsruhe, Germany) according to the manufacturer’s instructions. Cell culture media and untreated control samples were used for background normalization.

### Microarray analysis

Primary human macrophages were pre-incubated for 45 min with TLC (50 μM) and then stimulated with LPS (10 ng/ml) for 3 h or left untreated. Total RNA from cells was prepared with the ReliaPrep™ RNA Cell Miniprep System (Promega, Mannheim, Germany). Total RNA preparations were assessed for RNA integrity by Agilent 2100 Bioanalyzer (Santa Clara, California, USA) quality control. All samples in the present study showed high-quality RNA Integrity Numbers. RNA was further analysed by photometric Nanodrop measurement and quantified by fluorometric Qubit RNA assays (Life Technologies, Karlsruhe, Germany). To account for individual clinical sample-to-sample differences, equal amounts of total RNA from four donor samples per experimental condition were pooled prior to cRNA synthesis and array hybridization. Thus, 12 donor samples were analysed in three pooled replicates for each condition. Synthesis of cDNA and subsequent biotin labelling of cRNA was performed according to the manufacturer’s instructions (3′ IVT Plus Kit; Affymetrix, Inc., Karlsruhe, Germany). Briefly, 100 ng of pooled total RNA were converted to cDNA, followed by *in vitro* transcription and biotin labelling of cRNA. After fragmentation labelled cRNA was hybridized to Affymetrix PrimeView Human Gene Expression Microarrays for 16 h at 45 °C, stained by streptavidin/phycoerythrin conjugate and scanned according to the manufacturer’s protocol. Data analyses on Affymetrix CEL files were conducted with GeneSpring GX software (Vers. 12.5; Agilent Technologies). Probes within each probe set were summarized by Rate Monotonic Analysis (RMA) after quantile normalization of probe level signal intensities across all samples to reduce inter-array variability^58^. Input data pre-processing was concluded by baseline transformation to the median of all samples. To further improve signal-to-noise ratio, a given probe set had to be expressed above background (i.e., fluorescence signal of a probe set was detected within the 20th and 100th percentiles of the raw signal distribution of a given array) in all three replicates in at least one of two, or both conditions to be subsequently analysed in pairwise comparisons. Differential gene expression was statistically determined by moderated T-tests. If applicable, the resulting p-values were corrected for multiple testing (Benjamini-Hochberg FDR). The significance threshold was set to p = 0.01 or p_(corr)_ = 0.01, respectively. GeneOntology (GO) analyses were performed using the DAVID Functional Annotation Tool (http://david.abcc.ncifcrf.gov/) testing for enrichment of differentially expressed transcripts in distinct functional GO categories. Significant GO term enrichment was determined at an EASE score of p-value < 0.01.

### Real-time PCR

The mRNA expression was assessed by semi-quantitative PCR ((qPCR) GoTaq qPCR Master Mix; Promega) and was performed with the following primers for human: HPRT1_for: gctttccttggtcaggcagt; HPRT1_rev: gcttgcgaccttgaccatct; CCL3_for: attccgtcacctgctcagaa; CCL3_rev: gcagcaagtgatgcaga; CCL4_for: aagtctgtgctgatcccagt; CCL4_rev: caggtgaccttccctgaaga; CCL5_for: cagcagtcgtctttgtcacc; CCL5_rev: aggactctccatcctagctca; CXCL1_for: aaagcttgcctcaatcctgc; CXCL1_rev: ggtcagttggatttgtcactgt; CXCL2_for: atgtctttcttgtaaggcatactg; CXCL2_rev: cgaaacctctctgctctaacac; CXCL3_for: gcgtatcattgacacttcctgc; CXCL3_rev: cctttccagctgtccctagaa; CXCL9_for: ccaatacaggagtgacttggaac; CXCL9_rev: tcactactgggttccttgc; CXCL10_for: ctgagcctacagcagaggaa; CXCL10_rev: gagaggtactccttgaatgcc; CXCL11_for: agcaagcaaggcttataatcaaaaa; CXCL11_rev: ttgtcatttcagtagtcacagtta; MMP10_for: ggggaagacagatatgggtg; MMP10_rev: tgcaaggctcatcttcttca; MMP12_for: gacccaaaagagaaccaacg; MMP12_rev: tctcagaaaccttcagccag; IL15_for: aaacagaagccaactgggtg; IL-15_rev: ctttgcaactggggtgaaca; IL18_for: gctgaagatgatgaaaacctgg; IL18_rev: ggccgatttccttggtcaat; EREG_for: gcctgggtttccatcttcta; EREG_rev: cacacgtggattgtcttctg.

The qPCR was performed using SYBR Green Universal PCR Master Mix (Promega, Mannheim, Germany) with the ViiA7 Real-Time PCR System (Applied Thermo Fisher, Karlsruhe, Germany) in 96-well optical-reaction plates capped with optical adhesive covers (Applied Biosystems, Foster City, California, USA). The specificity of RT-PCR was controlled by adding no template and no reverse transcription controls. The results of qPCR were calculated using the ΔΔCt method.

### Statistical analysis

Results from different experiments are expressed as the means ± SEM (n = number of independent experiments; at least three). The results were compared using moderated two-tailed, equal variance study t-tests and Wilcoxon t-test. (*)P-value ≤ 0.05 and (**) ≤ 0.005 were considered statistically significant. Statistics were calculated using Prism 5.0 software from GraphPad Software (La Jolla, California, USA).

## Electronic supplementary material

Supplementary information

Data S1

Supplementary Data 2

Supplementary Data 3

## Data Availability

The microarray data reported in this paper are available from the NCBI database (https://www.ncbi.nlm.nih.gov/geo/query/acc.cgi?acc=GSE198326), and the real-time PCR data are included in Supplementary Data 2 and Supplementary Data 3. All other data used and analyzed during the current study are available from the corresponding author by reasonable request.
