# Author Correction: Reprogramming of pro-inflammatory human macrophages to an anti-inflammatory phenotype by bile acids

**DOI:** 10.1038/s41598-022-10306-9

**Published:** 2022-05-04

**Authors:** Marianne Wammers, Anna-Kathrin Schupp, Johannes G. Bode, Christian Ehlting, Stephanie Wolf, René Deenen, Karl Köhrer, Dieter Häussinger, Dirk Graf

**Affiliations:** 1 Department of Gastroenterology, Hepatology and Infectious Diseases, Heinrich-Heine-University Duesseldorf, Duesseldorf, Germany; 2 Biological and Medical Research Centre (BMFZ), Cluster of Excellence On Plant Sciences (CEPLAS), Heinrich-Heine-University Duesseldorf, Duesseldorf, Germany

Correction to: *Scientific Reports* 10.1038/s41598-017-18305-x, published online 10 January 2018

The original version of this Article was incomplete.

In the original version of the Supplementary Information, the log fold change values were not included. The original Supplementary Information is attached here.

In addition, Supplementary Data 2 and Supplementary Data 3 have been added, and the following Data Availability statement has been included,

“The microarray data reported in this paper are available from the NCBI database (https://www.ncbi.nlm.nih.gov/geo/query/acc.cgi?acc=GSE198326), and the real-time PCR data are included in Supplementary Data 2 and Supplementary Data 3. All other data used and analyzed during the current study are available from the corresponding author by reasonable request.”

The original Article and accompanying Supplementary Information files have been corrected.

## Supplementary Information

Supplementary Information.

