# Supplementary material for: Reprogramming of pro-inflammatory human macrophages to an anti-inflammatory phenotype by bile acids
Source: Sci Rep. 2018 Jan 10;8:255. doi: 10.1038/s41598-017-18305-x (PMC5762890; doi:10.1038/s41598-017-18305-x)
Supplement: Supplementary file 1 — Supplementary information [file 41598_2017_18305_MOESM1_ESM.pdf]

Supplementary Table S1: Distribution of transcripts analyzed in different pathways by KEGG.

Significantly expressed transcripts sorted in the different pathways by KEGG analysis. Regulated genes result from transcriptome analysis. Arrows show the up- or down-regulation of expressed transcripts or the unchanged expression by minus regarding the different conditions (LPS vs. LPS + TLC).

Term ID/ Term Name	Benjamini corrected p-value	Under stimulation of LPS	LPS + TLC	Regulated genes
KEGG_ hsa04060 Cytokine-cytokine receptor interaction	$1.9*10^{-19}$	↑	—	IL19, IL15, IL10, CLCF1, CCL3L1, CCL3L3, IL1B, IL15RA, CCL4L1, CCL4L2, CD40, IL20, OSM, CCR7, CCR5, VEGFA, IL12B, CSF3, CSF2, CCL3, CXCL5, CXCL2, IL7R, TNFRSF4, CCL4, LIF, IL23A, CCL23, CCL20, IL18R1, BMP2, IL8, CCL19, CCL15, TNFSF8, TSLP, TNFSF10, CCL14, IL3RA
		↑	↓	IL18, CXCL11, TNFSF18, TNFSF15, TNF, CXCL3, IL1A, CXCL10, TNFRSF9, CXCL1, CXCL9, CCL8, IL10RA, CCL5, TNFSF9, IL6
		↓	↑	KITLG, IL2RA
		↓	—	EPOR, TGFBR1, PLEKHO2, TNFRSF11A, CXCR4
KEGG_hsa04621 NOD-like receptor signalling pathway	$4.6*10^{-8}$	↑	—	IL8, RELA, CXCL2, NFKBIA, NFKB1, NLRP3, BIRC3, CASP5, NOD1, IL1B, TNFAIP3
		↑	↓	CXCL1, IL6, TNF, IL18, CCL8, CCL5, MEFV, RIPK2
		↓	—	NLRC4
KEGG_ hsa04620 Toll-like receptor signalling pathway	$6.8*10^{-8}$	↑	—	CCL3, IL8, RELA, TLR2, NFKBIA, NFKB1, CD40, MAP3K8, IRF7, RIPK1, TICAM1, CCL4, IL1B, IL12B
		↑	↓	CCL5, CXCL11, CXCL10, CD80, TNF, IL6, CXCL9
		↓	↓	FOS
		↓	—	PIK3CG, TLR5
KEGG_ hsa04623 Cytosolic DNA-sensing pathway	$9.6*10^{-6}$	↑	—	RELA, NFKBIA, CCL4L1, CCL4L2, NFKB1, CCL4, AIM2, DDX58, IRF7, RIPK1, IL1B, ADAR
		↑	↓	IL6, IL18, CCL5, CXCL10
KEGG_ hsa04622 RIG-I-like receptor signalling pathway	0,001	↑	—	IL8, RELA, NFKBIA, TRIM25, NFKB1DDX58, ISG15, RIPK1, IRF7, IL12B
		↑	↓	IFIH1, TNF, CXCL10, DHX58
KEGG_hsa04062 Chemokine signalling pathway	0,004	↑	—	CCL3, CXCL5, CXCL2, NFKBIA, NFKB1, CCL4, CCL23, CCL20, CCL3L1, CCL3L3, IL8, RELA, CCL19, CCL4L1, CCL4L2, CCL15, CCR7, CCL14, CCR5
		↑	↓	CXCL1, CXCL3, CXCL9, CXCL11, CCL8, CCL5, CXCL10
		↓	—	CXCR4, PIK3CG
KEGG_ hsa04660 T cell receptor signalling pathway	0,008	↑	—	RELA, NFKBIA, NFKB1, IL10, RASGRP1, MAP3K8, NFAT5, PPP3CC, CSF2
		↑	↓	TNF, LCP2
		↓	↓	FOS
		↓	—	PIK3CG, PTPN6, MAP3K14, NFATC3

The microarray data we used for KEGG-and GO-analysis are available from the NCBI database (https://www.ncbi.nlm.nih.gov/geo/query/acc.cgi?acc=GSE198326).

Next page

Supplementary Table S2: Distribution of transcripts analyzed in different pathways by GO.

Significantly expressed transcripts sorted in the different pathways by GO analysis. Regulated genes result from transcriptome analysis. Arrows show the up- or down-regulation of expressed transcripts or the unchanged expression by minus regarding the different conditions (LPS vs. LPS + TLC).

Supplementary Table S2: Distribution of transcripts analyzed in different pathways by GO pathways.

Term ID/ Term Name	Benjamini corrected p-value	Under stimulation of LPS	LPS + TLC	Regulated genes
GO:0006955 immune response	$1.7*10^{-24}$	↑	—	IL19, TLR2, TRGC2, IL15, IL10, CLEC4E, IL1B, CLEC4D, EBI3, GBP5, RELA, CCL4L1, CCL4L2, CCR7, CCR5, VEGFA, GBP3, CCL3, OAS3, OAS1, CCL4, IFI35, ADA, LIF, IL23A, CFB, IL1RN, AIM2, DDX58, TRAF3IP2, OASL, TNFSF10, APOL1, CD274, KYNU, IFI44L, NFKB2, CCL3L1, TICAM1, CCL3L3, ICAM1, FCAMR, OSM, CD83, IL12B, CSF3, CSF2, GPR183, , CXCL5, CXCL2, RSAD2, IL7R, TNFRSF4, CCL23, CCL20, , BCL2, TAP1, BCL3, PTX3, IL18R1, IL2RA, IL8, OLR1, CCL19, CCL15, TNFSF8, CCL14
		↑	↓	IL18, TNFSF15, CXCL11, TNFSF18, CXCL10, IL1A, IL27, HLA-F, GBP4, NBN, GBP2, GBP1, CCL8, IFIH1, OAS2, CCL5, DHX58, SLAMF7, XBP1, IRF8, RNF19B, GCH1, TNFSF9, CXCL3, CXCL9, TNF, LCP2, CXCL1, IL6
		↑	↑	BMP6, ETS1, NLRP3, SBNO2
		↓	↓	IL16, SMAD6
		↓	—	TLR5, CRTAM, BLNK, GPR65, CD180, MSH2, MBP, CXCR4
GO:0006954 inflammatory response	$3.1*10^{-22}$	↑	—	TLR2, NFKB1, IL15, IL10, NOD1, CD44, CCL3L1, HMOX1, TICAM1, CCL3L3, IL1B, NFKBIZ, RELA, CHST2, CCL4L1, CCL4L2, CD40, TNFAIP6, CCR7, CCR5, CCL3, ADORA2A, CXCL2, TNFRSF4, CCL4, IL23A, CCL23, CCL20, PTX3, B4GALT1, BMP2, IL2RA, OLR1, IL8, CFB, IL1RN, CCL19, IDO1, APOL2, APOL3, IRF7
		↑	↓	CXCL11, CXCL10, IL1A, IRAK2, F3, RIPK2, KDM6B, CXCL1, IL27, TNF, P2RX7, CXCL9, CCL8, CXCL3, IL6, CCL5
		↑	↑	FPR2, BMP6, ANXA1, NLRP3, MEFV, IGF2, INS-IGF2
		↓	↓	FOS
		↓	—	ADORA3, TLR5, NLRC4, CXCR4, NFATC3, BLNK, CD180
GO:0009611 response to wounding	$2.1*10^{-18}$	↑	—	INS-IGF2, TLR2, NFKB1, IL15, IL10, NOD1, CD44, CCL3L1, HMOX1, CCL3L3, TICAM1, IL1B, NFKBIZ, RELA, CHST2, CCL4L1, CCL4L2, CD40, PLAUR, TNFAIP6, CCR7, CCR5, ADM, CCL3, ADORA2A, CXCL2, FPR2, TNFRSF4, CCL4, IL23A, CCL23, CCL20, BCL2, PTX3, PLAT, B4GALT1, BMP2, IL2RA, OLR1, IL8, CFB, IL1RN, , CCL19, IDO1, SOD2, APOL2, CCNB1, PLSCR1, APOL3, IRF7, ID3
		↑	↓	CXCL10, CXCL11, SLC1A3, IL1A, IRAK2, F3, RIPK2, KDM6B, IL27, CXCL1, CXCL9, CCL8, TNF, CXCL3, CCL5, PLEK, IL6, P2RX7
		↑	↑	NLRP3, EREG, ITGB3, BMP6, ANXA1, MEFV, IGF2
		↓	↓	FOS
		↓	—	TLR5, NLRC4, CXCR4, NFATC3, BLNK, PTPN6, CD180
GO:0006952 defense response	$2.7*10^{-18}$	↑	—	KYNU, TLR2, NFKB1, IL15, IL10, CD48, NOD1, CD44, CCL3L1, HMOX1, CCL3L3, TICAM1, IL1B, MX1, FOSL1, NFKBIZ, RELA, CHST2, CCL4L1, CCL4L2, CD40, TNFAIP6, CD83, CCR7, CCR5, CCL3, ADORA2A, CXCL2, RSAD2, CCL4, TNFRSF4, IL23A, CCL23, CCL20, BCL2, TAP1, BCL3, PTX3, B4GALT1, IL18R1, BMP2, IL2RA, OLR1, IL8, CFB, IL1RN, CCL19, IDO1, DDX58, APOL2, APOL3, APOL1, IRF7
		↑	↓	P2RX7, SLAMF7, IL6, DHX58, CCL8, IL1A, IRAK2, CXCL10, CXCL11, F3, RIPK2, KDM6B, MX2, IL27, CXCL3, CXCL9, GCH1, TNF, CXCL1, IFIH1, CCL5
		↑	↑	BMP6, ANXA1, MEFV, NLRP3, INS-IGF2, FPR2, IGF2, TNIP1
		↓	↓	FOS
		↓	—	ADORA3, TLR5, NLRC4, CXCR4, SOCS6, BLNK, NFATC3, CD180
GO:0009615 response to virus	$9.4*10^{-12}$	↑	—	ZC3HAV1, RSAD2, CCL4, IFI35, ISG20, TRIM5, IL23A, ISG15, BCL2, TICAM1, BCL3, MX1, FOSL1, RELA, CCL19, CCL4L1, IFI44, CCL4L2, DDX58, PLSCR1, IRF7, EIF2AK2
		↑	↓	IL6, MX2, CCL8, CCL5, IFIH1, TNF
		↑	↑	NLRP3
		↑	—	CXCR4
GO:0001775 cell activation	$5.1*10^{-11}$	↑	—	CSF2, GPR183, ADORA2A, STAT5A, EDN1, TLR2, NFKB2, IL15, IL7R, TPD52, TNFRSF4, IL10, ADA, CD48, IL23A, CLCF1, BCL2, BCL11A, TICAM1, BCL3, RHOH, ICAM1, IL8, CD40, PLSCR1, IL12B
		↑	↓	NBN, TNF, LCP2, P2RX7, CD80, IRF1, RIPK2, SLAMF7, IL6, PLEK
		↑	↑	IRF4, SLAMF1, IGF2, SBNO2, INS-IGF2
		↓	↓	GAPT
		↓	—	SP3, BLNK, GIMAP1, CXCR4, MSH2, CRTAM
GO:0001817 regulation of cytokine production	$9.5*10^{-11}$	↑	—	PANX1, STAT5A, TLR2, NFKB1, TNFRSF4, IL10, NOD1, REL, HMOX1, TICAM1, IL1B, BCL3, EBI3, RELA, IGF2, IDO1, CD40, DDX58, CD83, EREG, IL12B
		↑	↓	TNF, IL1A, IL6, IL18, TNFSF15, RIPK2, CD80, IRF1, IL27, P2RX7
		↓	↑	INS-IGF2, NLRP3, IRF4
		↓	—	NLRP12, CRTAM, GIMAP1
GO:0045321 Leukocyte activation	$5.1*10^{-10}$	↑	—	CSF2, GPR183, STAT5A, EDN1, TLR2, IL15, IL7R, TPD52, TNFRSF4, IL10, ADA, CD48, IL23A, CLCF1, BCL2, BCL11A, TICAM1, BCL3, RHOH, ICAM1, IL8, CD40, IL12B
		↑	↓	IRF1, RIPK2, P2RX7, CD80, LCP2, SLAMF7, NBN
		↑	↑	INS-IGF2, SBNO2, IRF4, SLAMF1, IGF2
		↓	↓	GAPT
		↓	—	SP3, MSH2, CRTAM, BLNK, GIMAP1, CXCR4
