# Supplementary material for: Author Correction: Reprogramming of pro-inflammatory human macrophages to an anti-inflammatory phenotype by bile acids
Source: Sci Rep. 2022 May 4;12:7255. doi: 10.1038/s41598-022-10306-9 (PMC9068820; doi:10.1038/s41598-022-10306-9)
Supplement: Supplementary file 1 — Supplementary Information. [file 41598_2022_10306_MOESM1_ESM.pdf]

Reprogramming of pro-inflammatory human macrophages to an anti-inflammatory phenotype by bile acids.

Marianne Wammers[1#], Anna-Kathrin Schupp[1#], Johannes G. Bode[1], Christian Ehlting[1], Stephanie Wolf[1], René Deenen[2], Karl Köhrer[2], Dieter Häussinger[1], Dirk Graf[1*],

[1]Department of Gastroenterology, Hepatology and Infectious Diseases, Heinrich-Heine-University Duesseldorf, Germany

[2]Biological and Medical Research Centre (BMFZ), Cluster of Excellence on Plant Sciences (CEPLAS), Heinrich-Heine-University Duesseldorf, Germany

# These authors contributed equally.

Supplementary Table S1: Distribution of transcripts analyzed in different pathways by KEGG.

Significantly expressed transcripts sorted in the different pathways by KEGG analysis. Regulated genes result from transcriptome analysis. Arrows show the up- or down-regulation of expressed transcripts or the unchanged expression by minus regarding the different conditions (LPS vs. LPS + TLC).

Term ID/ Term Name	Benjamini corrected p-value	Under stimulation of LPS	LPS + TLC	Regulated genes
KEGG_ hsa04060 Cytokine-cytokine receptor interaction	$1.9*10^{-19}$	↑	—	IL19, IL15, IL10, CLCF1, CCL3L1, CCL3L3, IL1B, IL15RA, CCL4L1, CCL4L2, CD40, IL20, OSM, CCR7, CCR5, VEGFA, IL12B, CSF3, CSF2, CCL3, CXCL5, CXCL2, IL7R, TNFRSF4, CCL4, LIF, IL23A, CCL23, CCL20, IL18R1, BMP2, IL8, CCL19, CCL15, TNFSF8, TSLP, TNFSF10, CCL14, IL3RA
		↑	↓	IL18, CXCL11, TNFSF18, TNFSF15, TNF, CXCL3, IL1A, CXCL10, TNFRSF9, CXCL1, CXCL9, CCL8, IL10RA, CCL5, TNFSF9, IL6
		↓	↑	KITLG, IL2RA
		↓	—	EPOR, TGFBR1, PLEKHO2, TNFRSF11A, CXCR4
KEGG_hsa04621 NOD-like receptor signalling pathway	$4.6*10^{-8}$	↑	—	IL8, RELA, CXCL2, NFKBIA, NFKB1, NLRP3, BIRC3, CASP5, NOD1, IL1B, TNFAIP3
		↑	↓	CXCL1, IL6, TNF, IL18, CCL8, CCL5, MEFV, RIPK2
		↓	—	NLRC4
KEGG_ hsa04620 Toll-like receptor signalling pathway	$6.8*10^{-8}$	↑	—	CCL3, IL8, RELA, TLR2, NFKBIA, NFKB1, CD40, MAP3K8, IRF7, RIPK1, TICAM1, CCL4, IL1B, IL12B
		↑	↓	CCL5, CXCL11, CXCL10, CD80, TNF, IL6, CXCL9
		↓	↓	FOS
		↓	—	PIK3CG, TLR5
KEGG_ hsa04623 Cytosolic DNA-sensing pathway	$9.6*10^{-6}$	↑	—	RELA, NFKBIA, CCL4L1, CCL4L2, NFKB1, CCL4, AIM2, DDX58, IRF7, RIPK1, IL1B, ADAR
		↑	↓	IL6, IL18, CCL5, CXCL10
KEGG_ hsa04622 RIG-I-like receptor signalling pathway	0,001	↑	—	IL8, RELA, NFKBIA, TRIM25, NFKB1DDX58, ISG15, RIPK1, IRF7, IL12B
		↑	↓	IFIH1, TNF, CXCL10, DHX58
KEGG_hsa04062 Chemokine signalling pathway	0,004	↑	—	CCL3, CXCL5, CXCL2, NFKBIA, NFKB1, CCL4, CCL23, CCL20, CCL3L1, CCL3L3, IL8, RELA, CCL19, CCL4L1, CCL4L2, CCL15, CCR7, CCL14, CCR5
		↑	↓	CXCL1, CXCL3, CXCL9, CXCL11, CCL8, CCL5, CXCL10
		↓	—	CXCR4, PIK3CG
KEGG_ hsa04660 T cell receptor signalling pathway	0,008	↑	—	RELA, NFKBIA, NFKB1, IL10, RASGRP1, MAP3K8, NFAT5, PPP3CC, CSF2
		↑	↓	TNF, LCP2
		↓	↓	FOS
		↓	—	PIK3CG, PTPN6, MAP3K14, NFATC3

Next page

Supplementary Table S2: Distribution of transcripts analyzed in different pathways by GO.

Significantly expressed transcripts sorted in the different pathways by GO analysis. Regulated genes result from transcriptome analysis. Arrows show the up- or down-regulation of expressed transcripts or the unchanged expression by minus regarding the different conditions (LPS vs. LPS + TLC).

Supplementary Table S2: Distribution of transcripts analyzed in different pathways by GO pathways.

Term ID/ Term Name	Benjamini corrected p-value	Under stimulation of LPS	LPS + TLC	Regulated genes
GO:0006955 immune response	$1.7*10^{-24}$	↑	—	IL19, TLR2, TRGC2, IL15, IL10, CLEC4E, IL1B, CLEC4D, EBI3, GBP5, RELA, CCL4L1, CCL4L2, CCR7, CCR5, VEGFA, GBP3, CCL3, OAS3, OAS1, CCL4, IFI35, ADA, LIF, IL23A, CFB, IL1RN, AIM2, DDX58, TRAF3IP2, OASL, TNFSF10, APOL1, CD274, KYNU, IFI44L, NFKB2, CCL3L1, TICAM1, CCL3L3, ICAM1, FCAMR, OSM, CD83, IL12B, CSF3, CSF2, GPR183, , CXCL5, CXCL2, RSAD2, IL7R, TNFRSF4, CCL23, CCL20, , BCL2, TAP1, BCL3, PTX3, IL18R1, IL2RA, IL8, OLR1, CCL19, CCL15, TNFSF8, CCL14
		↑	↓	IL18, TNFSF15, CXCL11, TNFSF18, CXCL10, IL1A, IL27, HLA-F, GBP4, NBN, GBP2, GBP1, CCL8, IFIH1, OAS2, CCL5, DHX58, SLAMF7, XBP1, IRF8, RNF19B, GCH1, TNFSF9, CXCL3, CXCL9, TNF, LCP2, CXCL1, IL6
		↑	↑	BMP6, ETS1, NLRP3, SBNO2
		↓	↓	IL16, SMAD6
		↓	—	TLR5, CRTAM, BLNK, GPR65, CD180, MSH2, MBP, CXCR4
GO:0006954 inflammatory response	$3.1*10^{-22}$	↑	—	TLR2, NFKB1, IL15, IL10, NOD1, CD44, CCL3L1, HMOX1, TICAM1, CCL3L3, IL1B, NFKBIZ, RELA, CHST2, CCL4L1, CCL4L2, CD40, TNFAIP6, CCR7, CCR5, CCL3, ADORA2A, CXCL2, TNFRSF4, CCL4, IL23A, CCL23, CCL20, PTX3, B4GALT1, BMP2, IL2RA, OLR1, IL8, CFB, IL1RN, CCL19, IDO1, APOL2, APOL3, IRF7
		↑	↓	CXCL11, CXCL10, IL1A, IRAK2, F3, RIPK2, KDM6B, CXCL1, IL27, TNF, P2RX7, CXCL9, CCL8, CXCL3, IL6, CCL5
		↑	↑	FPR2, BMP6, ANXA1, NLRP3, MEFV, IGF2, INS-IGF2
		↓	↓	FOS
		↓	—	ADORA3, TLR5, NLRC4, CXCR4, NFATC3, BLNK, CD180
GO:0009611 response to wounding	$2.1*10^{-18}$	↑	—	INS-IGF2, TLR2, NFKB1, IL15, IL10, NOD1, CD44, CCL3L1, HMOX1, CCL3L3, TICAM1, IL1B, NFKBIZ, RELA, CHST2, CCL4L1, CCL4L2, CD40, PLAUR, TNFAIP6, CCR7, CCR5, ADM, CCL3, ADORA2A, CXCL2, FPR2, TNFRSF4, CCL4, IL23A, CCL23, CCL20, BCL2, PTX3, PLAT, B4GALT1, BMP2, IL2RA, OLR1, IL8, CFB, IL1RN, , CCL19, IDO1, SOD2, APOL2, CCNB1, PLSCR1, APOL3, IRF7, ID3
		↑	↓	CXCL10, CXCL11, SLC1A3, IL1A, IRAK2, F3, RIPK2, KDM6B, IL27, CXCL1, CXCL9, CCL8, TNF, CXCL3, CCL5, PLEK, IL6, P2RX7
		↑	↑	NLRP3, EREG, ITGB3, BMP6, ANXA1, MEFV, IGF2
		↓	↓	FOS
		↓	—	TLR5, NLRC4, CXCR4, NFATC3, BLNK, PTPN6, CD180
GO:0006952 defense response	$2.7*10^{-18}$	↑	—	KYNU, TLR2, NFKB1, IL15, IL10, CD48, NOD1, CD44, CCL3L1, HMOX1, CCL3L3, TICAM1, IL1B, MX1, FOSL1, NFKBIZ, RELA, CHST2, CCL4L1, CCL4L2, CD40, TNFAIP6, CD83, CCR7, CCR5, CCL3, ADORA2A, CXCL2, RSAD2, CCL4, TNFRSF4, IL23A, CCL23, CCL20, BCL2, TAP1, BCL3, PTX3, B4GALT1, IL18R1, BMP2, IL2RA, OLR1, IL8, CFB, IL1RN, CCL19, IDO1, DDX58, APOL2, APOL3, APOL1, IRF7
		↑	↓	P2RX7, SLAMF7, IL6, DHX58, CCL8, IL1A, IRAK2, CXCL10, CXCL11, F3, RIPK2, KDM6B, MX2, IL27, CXCL3, CXCL9, GCH1, TNF, CXCL1, IFIH1, CCL5
		↑	↑	BMP6, ANXA1, MEFV, NLRP3, INS-IGF2, FPR2, IGF2, TNIP1
		↓	↓	FOS
		↓	—	ADORA3, TLR5, NLRC4, CXCR4, SOCS6, BLNK, NFATC3, CD180
GO:0009615 response to virus	$9.4*10^{-12}$	↑	—	ZC3HAV1, RSAD2, CCL4, IFI35, ISG20, TRIM5, IL23A, ISG15, BCL2, TICAM1, BCL3, MX1, FOSL1, RELA, CCL19, CCL4L1, IFI44, CCL4L2, DDX58, PLSCR1, IRF7, EIF2AK2
		↑	↓	IL6, MX2, CCL8, CCL5, IFIH1, TNF
		↑	↑	NLRP3
		↑	—	CXCR4
GO:0001775 cell activation	$5.1*10^{-11}$	↑	—	CSF2, GPR183, ADORA2A, STAT5A, EDN1, TLR2, NFKB2, IL15, IL7R, TPD52, TNFRSF4, IL10, ADA, CD48, IL23A, CLCF1, BCL2, BCL11A, TICAM1, BCL3, RHOH, ICAM1, IL8, CD40, PLSCR1, IL12B
		↑	↓	NBN, TNF, LCP2, P2RX7, CD80, IRF1, RIPK2, SLAMF7, IL6, PLEK
		↑	↑	IRF4, SLAMF1, IGF2, SBNO2, INS-IGF2
		↓	↓	GAPT
		↓	—	SP3, BLNK, GIMAP1, CXCR4, MSH2, CRTAM
GO:0001817 regulation of cytokine production	$9.5*10^{-11}$	↑	—	PANX1, STAT5A, TLR2, NFKB1, TNFRSF4, IL10, NOD1, REL, HMOX1, TICAM1, IL1B, BCL3, EBI3, RELA, IGF2, IDO1, CD40, DDX58, CD83, EREG, IL12B
		↑	↓	TNF, IL1A, IL6, IL18, TNFSF15, RIPK2, CD80, IRF1, IL27, P2RX7
		↓	↑	INS-IGF2, NLRP3, IRF4
		↓	—	NLRP12, CRTAM, GIMAP1
GO:0045321 Leukocyte activation	$5.1*10^{-10}$	↑	—	CSF2, GPR183, STAT5A, EDN1, TLR2, IL15, IL7R, TPD52, TNFRSF4, IL10, ADA, CD48, IL23A, CLCF1, BCL2, BCL11A, TICAM1, BCL3, RHOH, ICAM1, IL8, CD40, IL12B
		↑	↓	IRF1, RIPK2, P2RX7, CD80, LCP2, SLAMF7, NBN
		↑	↑	INS-IGF2, SBNO2, IRF4, SLAMF1, IGF2
		↓	↓	GAPT
		↓	—	SP3, MSH2, CRTAM, BLNK, GIMAP1, CXCR4

Supplementary Table S3: LPS-induced immune genes regulated by TLC.

Immune regulatory genes presented in fig. 2. Gene symbols with gene titles are sorted by function.

Gene Symbol	Gene Title	Function
ALCAM	activated leukocyte cell adhesion molecule	activation, differentiation and migration
CCL5	chemokine (C-C motif) ligand 5	activation, differentiation and migration
CD80	CD80 molecule	activation, differentiation and migration
CXCL1	chemokine (C-X-C motif) ligand 1 (melanoma growth stimulating activity, alpha)	activation, differentiation and migration
CXCL1 / 2	chemokine (C-X-C motif) ligand 1/ 2 (melanoma growth stimulating activity, alpha)	activation, differentiation and migration
CXCL10	chemokine (C-X-C motif) ligand 10	activation, differentiation and migration
CXCL11	chemokine (C-X-C motif) ligand 11	activation, differentiation and migration
CXCL3	chemokine (C-X-C motif) ligand 3	activation, differentiation and migration
CXCL9	chemokine (C-X-C motif) ligand 9	activation, differentiation and migration
IL27	interleukin 27	activation, differentiation and migration
IL18	interleukin 18 (interferon-gamma-inducing factor)	activation, differentiation and migration
CCL8	chemokine (C-C motif) ligand 8	activation, differentiation and migration
KCNA3	potassium voltage-gated channel, shaker-related subfamily, member 3	activation, differentiation and migration
TNFRSF9	tumor necrosis factor receptor superfamily, member 9	activation, differentiation and migration
TNFSF18	tumor necrosis factor (ligand) superfamily, member 18	activation, differentiation and migration
TNFSF9	tumor necrosis factor (ligand) superfamily, member 9	activation, differentiation and migration
APOBEC3A /_B	apolipoprotein B mRNA editing enzyme, catalytic polypeptide-like 3A	direct interaction with pathogenes
ARL5B	ADP-ribosylation factor-like 5B	direct interaction with pathogenes
DHX58	DEXH (Asp-Glu-X-His) box polypeptide 58	direct interaction with pathogenes
GBP1	guanylate binding protein 1, interferon-inducible	direct interaction with pathogenes
GBP2	guanylate binding protein 2, interferon-inducible	direct interaction with pathogenes
IFIH1	interferon induced with helicase C domain 1	direct interaction with pathogenes
IFITM1	interferon induced transmembrane protein 1	direct interaction with pathogenes
MB21D1	Mab-21 domain containing 1	direct interaction with pathogenes
MX2	myxovirus (influenza virus) resistance 2 (mouse)	direct interaction with pathogenes
OAS2	2'-5'-oligoadenylate synthetase 2, 69/71kDa	direct interaction with pathogenes
IL10RA	interleukin 10 receptor, alpha	chemokine/cytokine signaling
IRAK2	interleukin-1 receptor-associated kinase 2	chemokine/cytokine signaling
IRF1	interferon regulatory factor 1	chemokine/cytokine signaling
SRC	v-src sarcoma (Schmidt-Ruppin A-2) viral oncogene homolog (avian)	chemokine/cytokine signaling
IL1A	interleukin 1, alpha	proinflammatory effects
IL6	interleukin 6 (interferon, beta 2)	proinflammatory effects
TNF	tumor necrosis factor	proinflammatory effects
HES1	hairy and enhancer of split 1, (Drosophila)	phatogen detection
JAG1	jagged 1	phatogen detection
RIPK2	receptor-interacting serine-threonine kinase 2	phatogen detection
KLF4	Kruppel-like factor 4 (gut)	phatogen detection
MST4	serine/threonine protein kinase MST4	phatogen detection
PELI1	pellino E3 ubiquitin protein ligase 1	phatogen detection
PML	promyelocytic leukemia	phatogen detection
STAP1	signal transducing adaptor family member 1	phatogen detection
XBP1	X-box binding protein 1	phatogen detection
ARID5A	AT rich interactive domain 5A (MRF1-like)	NO production
GCH1	GTP cyclohydrolase 1	NO production
P2RX7	purinergic receptor P2X, ligand-gated ion channel, 7	NO production
TNFSF15	tumor necrosis factor (ligand) superfamily, member 15	NO production
C10orf10	chromosome 10 open reading frame 10	phagocytosis and autophagy
IRF8	interferon regulatory factor 8	phagocytosis and autophagy
LDLR	low density lipoprotein receptor	phagocytosis and autophagy
NAMPT	nicotinamide phosphoribosyltransferase	phagocytosis and autophagy
ATF3	activating transcription factor 3	regulation of cytokine/chemokine expression
CISH	cytokine inducible SH2-containing protein	regulation of cytokine/chemokine expression
GBP4	guanylate binding protein 4	regulation of cytokine/chemokine expression
PRDM1	PR domain containing 1, with ZNF domain	regulation of cytokine/chemokine expression
PSTPIP2	proline-serine-threonine phosphatase interacting protein 2	regulation of cytokine/chemokine expression
SLAMF7	SLAM family member 7	regulation of cytokine/chemokine expression
SP110	SP110 nuclear body protein	regulation of cytokine/chemokine expression
ZC3H12C	zinc finger CCCH-type containing 12C	regulation of cytokine/chemokine expression

Immune regulatory genes presented in fig. 4. Gene symbols with gene titles are sorted by function.

Gene Symbol	Gene Title	Function
ANGPTL4	angiopoietin-like 4	Anti-inflammatory effects
ANXA1	annexin A1	Anti-inflammatory effects
IRF4	interferon regulatory factor 4	Anti-inflammatory effects
MEFV	Mediterranean fever	Anti-inflammatory effects
PTPN1	protein tyrosine phosphatase, non-receptor type 1	Anti-inflammatory effects
SBNO2	strawberry notch homolog 2 (Drosophila)	Anti-inflammatory effects
SLAMF1	signaling lymphocytic activation molecule family member 1	Anti-inflammatory effects
TNIP1	TNFAIP3 interacting protein 1	Anti-inflammatory effects
GADD45A	growth arrest and DNA-damage-inducible, alpha	chemotactic response
ADAMTS4	ADAM metallopeptidase with thrombospondin type 1 motif, 4	proliferation and differentiation
EREG	epiregulin	wound healing, proliferation and differentiation
VEGFA	vascular endothelial growth factor A	proliferation and differentiation
MTF1	metal-regulatory transcription factor 1	pro-inflammatory effects
BMP6	bone morphogenetic protein 6	pro-inflammatory effects
IL2RA	interleukin 2 receptor, alpha	pro-inflammatory effects
NLRP3	NLR family, pyrin domain containing 3	pro-inflammatory effects
STAT4	signal transducer and activator of transcription 4	pro-inflammatory effects
